# Exploring Components and Feasibility of Health Coaching Interventions for Self-Management of Type 2 Diabetes: Protocol for a Systematic Review

**DOI:** 10.2196/71383

**Published:** 2026-03-31

**Authors:** Rija Mir, Shoba Poduval, Jessica Sheringham, Fiona Louise Hamilton

**Affiliations:** 1 Research Department of Primary Care and Population Health, University College London, Upper Third Floor, UCL Medical School (Royal Free Campus), Rowland Hill Street, London, United Kingdom, 44 2080168013; 2 Centre for Primary Care, Wolfson Institute of Population Health, Queen Mary University of London, London, United Kingdom

**Keywords:** health coaching, digital health interventions, eHealth, type 2 diabetes, T2D, self-management, digital divide, remote coaching, RC, virtual coaching, mobile health

## Abstract

**Background:**

Type 2 diabetes (T2D) currently has no cure. However, extensive evidence suggests that addressing key risk factors through lifestyle changes can help individuals effectively self-manage their condition. Diabetes self-management primarily involves patients engaging in self-monitoring behaviors and adopting coping strategies to manage their long-term illness. In recent years, health coaching interventions have gained recognition as a valuable approach for providing personalized support, enabling patients to take an active role in managing their health. By fostering behavior change through goal setting, active learning, accountability, and empowerment, health coaching equips patients with the tools to proactively manage their condition over time. This approach is especially important for researchers and policymakers, as it underscores the need for acceptable, engaging, and personalized care interventions that have a lasting positive impact, promoting self-sufficiency and improved quality of life for individuals with T2D.

**Objective:**

The objective of this study is to systematically review published, peer-reviewed, primary research studies to evaluate the effectiveness of health coaching interventions for T2D self-management, with glycated hemoglobin (HbA_1c_) as the primary outcome. It aims to examine how intervention components, delivery modalities, and health coach characteristics relate to effectiveness, acceptability, and engagement.

**Methods:**

This study protocol outlines the procedure for a systematic review that follows the methodology recommended by PRISMA-P (Preferred Reporting Items for Systematic Reviews and Meta-Analyses Protocols) guidelines. The review will include randomized controlled trials, quasi-experimental studies, and qualitative studies published in English up to December 1, 2024. The following databases will be used to conduct searches: MEDLINE, PsycINFO, Embase, CINAHL, Web of Science, and the Cochrane Central Register of Controlled Trials, with additional studies identified through citation searching and reference list screening. Included studies will consist of health coaching interventions delivered one-to-one through face-to-face, remote or digital, or artificial intelligence–supported formats. The primary outcome of interest is HbA_1c_, alongside additional clinical outcomes and qualitative data related to acceptability and engagement. Study selection will involve independent screening by reviewers, with disagreements resolved by consensus. Quantitative data will be synthesized using narrative synthesis, and qualitative findings will be analyzed using thematic and framework analysis within a staged, segregated synthesis approach.

**Results:**

As of September 2025, database searches have been conducted, and decisions have been made related to the included publications. Data extraction and analysis are currently ongoing. Results are expected to be published in summer 2026. The review is underway and is anticipated to be completed by May 2026.

**Conclusions:**

This review will synthesize evidence on the effectiveness and implementation of health coaching interventions for T2D and is expected to inform the design of effective and sustainable coaching models for diabetes self-management.

## Introduction

### Type 2 Diabetes

Recent statistics from the World Health Organization (WHO) show that approximately 41 million people globally die of a chronic illness each year, of which 2 million are attributed to diabetes mellitus alone [1]. Currently, while more than half a billion people live with this condition, with 96% of these cases identified as type 2 diabetes (T2D), these numbers are projected to double in the next 30 years, estimating an increase in global diabetes-related health expenditure to US $1054 billion by 2045 [2,3]. T2D occurs when the body is unable to regulate blood glucose levels because it is either resistant to the insulin being produced or the pancreas does not make enough insulin. Living with this lifelong chronic condition not only affects individuals and their families, but associated risks, such as morbidity, premature mortality, mental health problems, hospitalizations, medications, and other medical costs, also significantly contribute to the overall global burden of T2D on health care systems [4]. It has also been predicted that T2D will be a serious clinical and financial challenge to the UK National Health Service for the next 6 decades, with a significant increase in patient caseloads and the population burden for diabetes-related complications [5].

There is no cure for T2D yet; however, extensive evidence indicates that minimizing several risk factors through lifestyle changes can help people self-manage their condition [6-8]. Self-management in diabetes primarily consists of patients undertaking self-monitoring behaviors and activities or using strategies to cope with their long-term illness. These include frequent monitoring of blood glucose levels, maintaining a healthy daily intake of nutrition, adherence to prescribed medication, insulin dose adjustments, and regular physical activity [9]. Self-management not only empowers patients to make informed decisions and ultimately achieve their health goals but also helps prevent further complications, reducing the strain on health care services and the overall burden of disease [10]. Prior study findings have significantly established that self-management of T2D improves clinical health outcomes and well-being through an overall positive impact on patient health behaviors, such as engaging in moderate physical activity, consuming a low glycemic index diet, and adhering to medication, among others [11-14]. Thus, lifelong proactive diabetes self-management is crucial for T2D care.

### Health Coaching

More recently, lifestyle interventions, such as health coaching, have gained increasing recognition as an effective strategy for providing personalized support to enable patients to actively self-manage their health conditions [15]. Rooted in health education and counseling psychology, health coaching adopts a one-on-one, personalized approach that prioritizes patient-centered care [16-19]. It supports patients in learning how to proactively self-manage their condition throughout their lives, by promoting behavior change through goal setting, active learning processes, accountability, and patient empowerment [20,21]. Central to health coaching is the relationship cultivated between the coach and patient through multiple interactions, which significantly influences the patient’s understanding, learning, behavior, and progress [22]. Substantial study findings confirm that health coaching interventions have shown a significant positive impact on clinical and behavioral outcomes in patients with T2D [23-27]. However, some systematic reviews have found mixed results, with little to no significant improvement in some clinical outcomes and other health outcomes, such as quality of life, due to self-management [28,29]. Several reasons have been attributed to the mixed evidence in current literature, such as the heterogeneity of the interventions, imprecise descriptions of interventions, and lack of consensus or guidance on the components of a health coaching practice, resulting in variations in the reporting of outcomes [13]. Inconsistencies in the current literature regarding the effectiveness of health coaching interventions justify the need for further in-depth review to aid in our understanding and to identify factors contributing to their impact.

### Components of Health Coaching

There is a lack of standardization of how “health coach” or “health coaching” is defined [28]. Numerous definitions of health coaching are used in the current literature and there is an absence of a standardized strategy for coaching practice. Evidence from many studies highlights that health coaches are described as “educator,” “navigator,” “facilitator,” or “partner,” indicating that there is no clear definition of the role of a health coach, thus warranting critical analysis to establish key characteristics of a health coach. A health coach is central to a health coaching intervention because they are responsible for providing an effective coaching context where they influence a patient’s thoughts and behaviors to subsequently achieve their health goals. There is a strong need to systematically review key ingredients of a standard health coaching practice, such as theoretical underpinnings, the type of content to be included in the intervention, and strategies used to deliver the intervention, to determine the effectiveness of a health coaching intervention. At present, there is no consensus in the current literature on how to design an effective health coaching intervention that includes the most appropriate active components for behavior change [16]. Therefore, a focused systematic review will help identify which coaching practices are more appropriate and effective to replicate and use; this includes critically analyzing the components that make up the intervention (duration, frequency, and mode of delivery of sessions). Consequently, it will help identify factors contributing to effective, personalized, and engaging interactions with patients within a coaching context.

In addition, previous reviews have identified a paucity of research investigating specific health coaching program components in-depth [26,30]. For example, 1 aspect that remains underexplored but holds potential in evaluating the effectiveness of a health coach for T2D self-management is the delivery modality used to convey information to the patient [22]. This refers to the methods used for interacting with the patient, including (1) “face-to-face” (FF) coaching sessions, where both the coach and the individual are physically present in the same location during the session; (2) “remote” coaching (RC; also known as digital or tele coaching), conducted online without in-person contact through phone calls, video calls, SMS text messaging, emails, mobile apps, and similar means that rely on technology like mobile phones, computers, tablets, and other smart devices; and (3) “virtual coaching or artificial intelligence (AI)–supported coaching,” which uses autonomous systems or software to coach patients, such as AI coaches, e-coaches, virtual assistants, chatbots, and so on [31]. Previous literature has primarily examined and compared FF with RC health coaching, either exclusively or in combination, highlighting potential research gaps in fully examining and comparing interventions based on all 3 delivery modalities, as well as their combinations, for patients with T2D [32,33]. Accordingly, the literature recommends examining individual coaching practices and intervention components to clarify their role in intervention effectiveness and to inform future intervention design [34]. These include coaching techniques, intervention delivery modalities, frequency, and duration to determine how these features influence clinical and patient outcomes. Given that glycated hemoglobin (HbA_1c_) levels in patients with T2D are a strong indicator of disease management, understanding how each intervention component affects these levels is also integral and warranted.

### Technology-Enabled Health Coaching

Health coaching interventions for T2D are increasingly delivered through technology-enabled formats, including remote digital platforms and emerging AI-supported systems. The rapid expansion in the use of digital health technologies, such as eHealth and mobile health, has been largely accelerated by the COVID-19 pandemic, resulting in the widespread adoption of remote models of care within primary health care settings [35-37]. These developments have facilitated greater flexibility in service delivery and supported ongoing self-management for people with chronic conditions, including T2D [12,36,38]. As a result, technology-enabled health coaching has become an important modality for delivering personalized behavioral support.

Evidence suggests that remote health coaching interventions may improve access and convenience and have the potential to support behavior change; however, findings regarding their effectiveness remain inconsistent [39-41]. While some studies report positive impacts on self-management and clinical outcomes, others indicate limited benefits when compared with FF coaching, particularly with respect to sustaining engagement and behavior change over time [40-42]. These mixed findings highlight the importance of examining clinical effectiveness alongside intervention components and delivery characteristics when synthesizing outcomes such as HbA_1c_ across studies.

The expansion of digital health has also drawn attention to contextual factors that may influence patient experiences, particularly inequalities in access to and use of digital technologies, commonly described as the digital divide [43-47]. Evidence indicates that older adults, individuals from minority ethnic groups, and those from lower socioeconomic backgrounds may be disproportionately affected by limited digital access and literacy, which may shape engagement with remote or technology-based coaching interventions [48-52]. Although these factors will not be considered as primary outcomes when reviewed, they provide important context for interpreting patient-reported findings related to acceptability and engagement.

In parallel, growing scientific and policy interest in the application of AI within health care has led to the emergence of AI-supported coaching tools, including chatbots and virtual assistants, designed to support self-management behaviors [53]. However, existing systematic reviews have largely focused on FF and RC models, with limited examination of AI-based approaches [32,34]. Thus, this review will examine FF, remote or digital, and AI-supported health coaching interventions and synthesize HbA_1c_ outcomes alongside intervention components and contextual influences, enabling a comparative assessment of delivery modalities relevant to T2D self-management.

### Acceptability and Engagement

Although clinical outcomes, such as HbA_1c_ levels, have commonly been used to assess the effectiveness of digital health interventions, their real-world success largely depends on patients finding these interventions acceptable and maintaining engagement over time. Acceptability refers to an individual’s willingness to initiate and continue using an intervention [54]. This willingness is shaped by personal factors, such as beliefs, prior knowledge, and attitudes, as well as broader social and contextual influences [55]. Consequently, acceptability cannot be assumed to be uniform across populations, as it varies according to individual needs, experiences, and circumstances.

Engagement, often operationalized through quantitative metrics such as frequency or duration of use, is similarly multifaceted[56]. As Nicholas et al [57] argue, such measures may fail to capture key cognitive, emotional, and social determinants of engagement, including motivation, perceived relevance, and the availability of support. Qualitative evidence further demonstrates that the interplay between intervention content, user experience, and environmental context can either facilitate or hinder sustained participation. Thus, it is crucial to assess the acceptability and engagement of an intervention to determine its effectiveness and long-term sustainability.

Theoretical frameworks offer additional insight into mechanisms assessing acceptability and engagement as key indicators to determine the effectiveness of an intervention. Drawing on the Unified Theory of Acceptance and Use of Technology [58], there are 4 central determinants of technology uptake: perceived benefit, ease of use, social influence, and availability of support with moderators such as age, gender, and prior technology experience further influencing user behavior [59]. Similarly, behavior change interventions are more likely to be accepted when they enable users to establish personally meaningful goals, receive tailored feedback, and understand the rationale for recommended behaviors [60]. Empirical evidence supporting these propositions remains limited, indicating the need for further research to determine whether theory-driven designs consistently enhance engagement and clinical outcomes.

Perski and Short [55] emphasized the interdependence of acceptability, engagement, and effectiveness, positing that initial willingness to adopt an intervention influences ongoing engagement, which subsequently affects clinical results. Within diabetes care, engagement has been identified as a critical component of effective self-management [61], with features such as access to a health coach, digital monitoring tools, and personalized feedback contributing to sustained participation [62,63]. However, findings across studies remain mixed; some suggest that supplementing digital programs with FF support may reduce subsequent digital engagement [64], whereas others report minimal effects of RC on long-term use [65].

Given the increasing integration of digital and AI-enabled coaching within diabetes self-management, reliance solely on clinical outcomes provides an incomplete assessment of intervention success. Interventions that achieve improvements in HbA_1c_ but are perceived as unacceptable or fail to engage users are unlikely to be effective in routine practice. Furthermore, structural barriers associated with the digital divide, including socioeconomic constraints, cultural factors, and disparities in digital literacy, may limit uptake among certain populations [48-52]. Accordingly, a systematic review that examines not only effectiveness but also patient acceptability and engagement is essential. Such an approach will enable the identification of intervention components and delivery modalities most conducive to promoting long-term, equitable self-management for individuals with T2D.

Moreover, this holds significant importance for researchers and policymakers, ensuring that personalized care and health coaching interventions introduced to patients are sustainable and have a lasting positive impact on their lives, fostering self-sufficiency in managing their health condition. To the best of our knowledge, no prior systematic reviews have examined the acceptability, engagement, and effectiveness of health coaching interventions exclusively for T2D self-management. Therefore, this systematic review will aim to explore in-depth the contextual factors associated with the acceptability and engagement of health coaching interventions, which will aid in better understanding of the most effective intervention components. The findings obtained from studying acceptability and engagement will also help identify barriers and facilitators, offering a set of recommendations aimed at enhancing the effectiveness of health coaching interventions.

### Aims and Objectives

#### Aims

The primary aim of this systematic review is to evaluate the effectiveness of health coaching interventions for T2D self-management, with glycemic control (HbA_1c_) as the primary outcome. The review also aims to identify and compare intervention components, health coach characteristics, and delivery modalities associated with improved HbA_1c_ outcomes and to draw inferences regarding the feasibility and sustainability of different coaching approaches based on reported outcomes and intervention characteristics. Findings from this review will be used to inform recommendations for the design of effective and sustainable health coaching interventions for adults with T2D. This review seeks to address key research questions: (1) To what extent do health coaching interventions improve HbA_1c_ levels among adults with T2D compared with standard care or control conditions? (2) Which intervention components and health coach characteristics are associated with greater improvements in HbA_1c_ outcomes across included studies? (3) Which intervention components are most frequently present in effective health coaching interventions, and how do these component patterns vary across delivery modalities? (4) What insights do the included studies provide regarding the feasibility and sustainability of different delivery modalities, as inferred from HbA_1c_ outcomes and intervention characteristics?

#### Objectives

Drawing on existing evidence, this systematic review aims to (1) evaluate the effectiveness of health coaching interventions on glycemic control, with HbA_1c_ as the primary outcome, compared with standard care or control conditions; (2) identify intervention components and health coach characteristics associated with greater improvements in HbA_1c_ outcomes; and (3) compare delivery modalities (FF, remote or digital, and AI-supported coaching) in relation to effectiveness, feasibility, and sustainability, based on HbA_1c_ outcomes and intervention characteristics.

## Methods

This protocol follows the PRISMA-P (Preferred Reporting Items for Systematic Review and Meta-Analysis Protocols) statement (Checklist 1) [66]. This protocol has been registered with PROSPERO (CRD42025637862).

### Eligibility Criteria

#### Types of Studies

We will include experimental (randomized controlled trial), quasi-experimental, and qualitative study designs, which focus on in-depth interviews and focus group discussions, published until December 1, 2024.

The systematic review will include primary evidence from studies published in English that have high psychometric properties and methodological approaches used to minimize bias. Thus, the review will exclude observational studies, case reports, case series, letters to the editor, preprint papers, expert opinions, editorials, and narrative reviews (if a narrative review is considered relevant to the study, the list of included studies for that paper will be examined to identify studies that may have been missed during the search), abstract-only studies, conference proceedings, and study protocols.

#### Types of Participants

Studies with adult participants aged 18 years and older, having a confirmed diagnosis of type 2 diabetes or maturity-onset diabetes of the young, will be included. Studies with participants having type 1 diabetes or gestational diabetes will be excluded.

#### Types of Interventions

The review will include studies of health coaching interventions that provide one-on-one coaching delivered FF, remotely via digital technology, and virtually through autonomous systems or AI to individuals within a coaching context comprising a health coach.

#### Types of Comparators

The review will include studies with comparator interventions, such as (1) treatment as usual (standard care, leaflets, and health education programs) and (2) health interventions without one-on-one coaching, such as in groups. Studies with pretest or posttest study designs with no comparator at all will also be included.

#### Types of Outcome Measures

The review will include studies that report HbA_1c_ as an outcome measure related to T2D self-management. The study evaluations must include HbA_1c_ outcomes, and any additional clinical outcomes reported in these studies, such as fasting plasma glucose, lipid profiles, body weight, BMI, waist circumference, blood pressure, or urinary albumin measurements, will also be included where available. The review will also consider any additionally reported qualitative or patient-reported outcomes in these studies, such as facilitators, barriers, acceptability, uptake, or engagement relating to health coaching interventions.

### Search Strategy

The search strategy for the systematic review will aim to focus on both published and unpublished literature. An initial search of the databases will be conducted to identify research papers on the topic area. The titles and abstracts of relevant studies, as well as the keywords or index terms used to describe the studies, will be reviewed, and the most relevant terms will be selected to perform a full search using the following databases:

- MEDLINE (via Ovid, 1946 to December 1, 2024)
- PsycINFO (via Ovid, 1806 to December 1, 2024)
- Embase (via Ovid, 1947 to December 1, 2024)
- CINAHL (via EBSCOhost)
- Web of Science (core collection)
- Cochrane Central Library

These databases were selected to ensure comprehensive coverage of biomedical, nursing, behavioral, and digital health literature relevant to health coaching interventions for T2D. Search terms will include various synonyms, spellings, and acronyms for each identified keyword and index term. For example, for the MEDLINE database, the search will include MEDLINE thesaurus MeSH (Medical Subject Headings) terms as well as appropriate keywords related to “type 2 diabetes,” “health coaching interventions,” “remote coaching,” “e-coaching,” “virtual coaching,” “self-care,” “self-management,” “modalities,” “clinical outcomes,” “acceptability,” “engagement,” and “patient reported outcomes” (Multimedia Appendix 1). Similarly, the authors will perform all database searches in collaboration with a research librarian, and the search strategy will be adapted for each included information source. To focus and structure the search for optimal results, various search functions such as thesaurus, Boolean operators, abstract or title or keywords, phrase, free text, and advanced search will be used.

To identify any additional studies that did not appear during database searches, citation searches, and manual searches of the reference lists of all studies selected for critical appraisal will be performed. Moreover, citation searches will be conducted in Google Scholar and Web of Science. If it is difficult to access a study or if any questions arise during the selection or extraction processes, the authors of identified articles may be contacted. All bibliographic data from the search results will be transferred to EndNote (Clarivate), a reference information system software, and the finalized results will be collated and extracted to Microsoft Excel.

### Study Selection Process

One author (RM) will be responsible for conducting the database search and managing retrieved records. Following the removal of duplicates, all titles and abstracts identified through the search will be independently screened by at least 2 reviewers against the predefined inclusion criteria. Any records that do not meet the eligibility criteria will be excluded, with reasons for exclusion documented. To ensure consistency in the application of the inclusion criteria, reviewers will first pilot the screening process on an initial subset of studies and discuss discrepancies prior to full screening. All remaining titles and abstracts will then undergo independent double screening, and disagreements will be resolved through discussion and consensus.

Full-text studies of potentially eligible studies will be retrieved and independently assessed by at least 2 reviewers to confirm eligibility. Any disagreements at the full-text stage will be resolved through discussion, with the involvement of a third reviewer if necessary. The study selection process will be documented using a PRISMA (Preferred Reporting Items for Systematic Reviews and Meta-Analyses) flow diagram, detailing the number of records identified, screened, excluded, and included in the review.

### Data Collection Process and Data Extraction

The studies selected for full-text review will be independently examined, and the relevant data will be collected and recorded in a Microsoft Excel file. The data extracted will include specific details about the population, study methods, interventions, and outcomes of significance based on the review objective. More specifically, categories that are considered relevant include characteristics of the health coaching intervention and key results, characteristics of the health coach, and baseline characteristics of the population (Multimedia Appendix 1). The first author will conduct a full-text review of any 5 randomly selected studies and share them with the other authors to independently review and assess whether all the relevant information has been accurately extracted and meets the inclusion criteria. The feedback will be discussed, and any disagreements that arise between the reviewers will be resolved through discussion or with a third or additional reviewer. The author will then continue to review and extract data from the remaining studies based on their feedback.

### Outcomes and Prioritization

The primary outcome of interest for this review is HbA_1c_, reported as a validated clinical measure of glycemic control in adults with T2D. HbA_1c_ outcomes will be extracted as reported in the included studies, including the timing of outcome assessment. Where available, additional clinical outcomes will also be extracted, including fasting plasma glucose, lipid profiles, body weight, BMI, waist circumference, blood pressure, and urinary albumin measurements.

In addition to clinical outcomes, the review will include studies reporting qualitative or patient-reported outcomes related to the use of health coaching interventions. These outcomes will focus on acceptability and engagement, examined using qualitative data and patient-reported findings as defined in the original studies. Acceptability will be interpreted as patients’ reported willingness to use, perceived suitability of, and satisfaction with the intervention. Engagement will be interpreted based on reported patterns of use, interaction with the intervention or coach, and sustained participation over time. Data relating to facilitators, barriers, uptake, and user experiences will be synthesized narratively to contextualize and interpret variation in intervention effectiveness across delivery modalities and intervention components.

### Risk of Bias and Quality Assessment

Risk of bias and methodological quality of the included studies will be independently assessed by at least 2 reviewers using design-appropriate appraisal tools. Randomized controlled trials will be evaluated using the Cochrane Collaboration’s Risk of Bias tool, nonrandomized quantitative studies will be assessed using the ROBINS-I (Risk of Bias in Nonrandomized Studies of Interventions) tool, and qualitative studies will be appraised using the CASP (Critical Appraisal Skills Program) checklist (Checklist 2) [67-69].

To ensure the consistent application of bias assessment criteria, reviewers will pilot the appraisal tools on a subset of included studies and discuss discrepancies prior to completing assessments for all studies. Any disagreements arising during the risk-of-bias assessment will be resolved through discussion and consensus.

All studies will be included in the data extraction and synthesis regardless of their assessed risk of bias. However, risk-of-bias judgments will be taken into account during the interpretation of the findings, with greater caution applied when interpreting results from studies assessed as having a high risk of bias. The potential influence of study quality on observed patterns of effectiveness will be explored narratively as part of the synthesis. Where necessary, authors of the included studies may be contacted to clarify methodological details or to obtain missing information relevant to the risk-of-bias assessment. In addition, the overall quality and certainty of the body of evidence may be assessed using the grading of recommendations, assessment, development, and evaluations (GRADE) approach, where appropriate [70].

### Data Synthesis, Narrative Synthesis, or Meta-Analysis

All included studies will be reviewed in depth, and relevant data will be extracted and synthesized in accordance with the review objectives and research questions. Given the inclusion of both quantitative and qualitative study designs, a segregated, staged synthesis approach will be used, whereby quantitative and qualitative evidence will first be analyzed separately using methods appropriate to each data type, followed by an integrative interpretive stage.

For quantitative studies, changes in HbA_1c_ levels will be synthesized using narrative synthesis, structured around study design, intervention characteristics, delivery modality, and effectiveness relative to comparator conditions. Meta-analysis will be considered where sufficient homogeneity exists in study design, intervention characteristics, and outcome reporting. However, substantial heterogeneity across interventions and outcome measures may preclude statistical pooling.

For qualitative studies, data relating to acceptability, engagement, barriers, and facilitators will be analyzed using thematic analysis, followed by framework analysis. An initial analytical framework will be developed based on existing theory and prior research, including the Bandura [71] reciprocal determinism theory, and will be sufficiently flexible to accommodate inductively derived themes. Line-by-line coding will be conducted to generate initial codes, which will be organized into higher-order themes and subthemes [72]. The framework will be iteratively refined by integrating emerging themes and restructuring categories as required to ensure comprehensive representation of the data. Qualitative analyses will be reviewed by multiple authors, with discrepancies resolved through discussion and consensus. Formal double-coding, use of qualitative analysis software, and calculation of inter-rater reliability statistics (eg, κ) are not planned, given the interpretive nature of the thematic and framework analysis; instead, credibility will be ensured through reviewer discussion and consensus.

In the final stage, findings from the quantitative and qualitative syntheses will be integrated interpretively. Qualitative themes will be used to contextualize and explain patterns observed in quantitative outcomes, particularly variations in HbA_1c_ effectiveness across intervention components and delivery modalities. This staged integration will support the development of a conceptual framework identifying how intervention characteristics, delivery strategies, and contextual factors interact to influence effectiveness, acceptability, and engagement with health coaching interventions for T2D self-management.

Heterogeneity in intervention design, delivery modality, follow-up duration, and outcome measurement is anticipated. HbA_1c_ outcomes will be extracted and synthesized as reported in the included studies, with the timing of measurement documented to support interpretation. To accommodate variability across studies, quantitative findings will be synthesized using narrative synthesis, and meta-analysis will only be undertaken where outcome measures and time points are sufficiently comparable. Where sufficient data are available, descriptive subgroup analyses will be conducted by delivery modality (FF, remote or digital, and AI-supported interventions) and by key intervention components to explore potential sources of heterogeneity. Formal sensitivity analyses will not be undertaken due to anticipated variability in study design, intervention characteristics, and outcome measurement; instead, the robustness of findings will be assessed through structured comparative narrative synthesis across subgroups.

## Results

The systematic review is in progress, and the final draft is anticipated to be completed by May 2026. We expect to report a summary of the findings of included published and unpublished studies that involve the delivery and use of health coaching interventions for patients to self-manage their T2D within a coaching context, including changes in HbA_1c_ levels and other clinical outcomes, as well as the results of qualitative studies that identify the barriers, facilitators, and challenges associated with the use and feasibility of health coaching interventions by patients with T2D. Database searches have been conducted, and decisions have been made related to the included publications. Data extraction and analysis are currently ongoing. Results are expected to be published in summer 2026.

## Discussion

### Principal Findings

The systematic review seeks to synthesize from existing evidence the impacts of using health coaching interventions on glycemic control in adults with T2D. It is anticipated that interventions incorporating structured goal setting, personalized feedback, regular coach-patient interaction, and clearly defined coaching roles will be more consistently associated with favorable HbA_1c_ outcomes. The review also aims to identify differences in effectiveness and feasibility across FF, remote or digital, and AI-supported coaching formats, reflecting the diversity of delivery approaches currently used in practice.

This review is expected to extend existing evidence by synthesizing findings across multiple delivery modalities and explicitly linking HbA_1c_ outcomes to intervention components and health coach characteristics. By adopting a component-focused synthesis, the review aims to provide a more nuanced understanding of how and why certain coaching interventions may be more effective than others.

In addition, by integrating qualitative findings on barriers, facilitators, acceptability, and engagement, this review will contextualize quantitative effectiveness data and support the interpretation of variation in outcomes. This approach is particularly relevant given the growing reliance on remote and technology-enabled coaching interventions, where patient engagement and contextual factors may influence real-world effectiveness.

A key strength of this review is its inclusive and structured synthesis approach, combining quantitative and qualitative evidence using a staged, segregated design. This enables clinical effectiveness (HbA_1c_) to be prioritized while also incorporating patient-reported experiences relevant to acceptability, engagement, feasibility, and sustainability. The inclusion of FF, remote or digital, and AI-supported interventions allows for a comprehensive comparison across delivery modalities that have not been examined in prior reviews [32-34].

However, several limitations are anticipated. Heterogeneity in intervention design, outcome measurement, follow-up duration, and reporting practices may restrict the feasibility of a meta-analysis, thereby requiring reliance on narrative synthesis. Additionally, acceptability and engagement are expected to be variably defined and reported across studies, which may constrain direct comparisons. Risk-of-bias assessments will, therefore, be used to inform interpretation, and conclusions will be drawn cautiously where evidence quality is limited.

In addition to these methodological limitations, several practical constraints of the planned review may introduce potential bias. The restriction to English-language publications may result in language bias and the exclusion of relevant studies conducted in non–English-speaking settings. Furthermore, the exclusion of gray literature, such as reports, theses, and unpublished studies, may increase the risk of publication bias by preferentially capturing studies with positive or statistically significant findings. These constraints, though common, may limit the comprehensiveness and generalizability of the evidence base. Findings from this review will, therefore, be interpreted with these considerations in mind, and gaps arising from these exclusions will be highlighted to inform future research.

The findings of this review are expected to inform future intervention design by identifying which health coaching components and delivery approaches are most consistently associated with improved glycemic control. For researchers, the synthesis will highlight gaps in reporting and areas where standardization of intervention descriptions and outcome measures is needed. For practitioners and policymakers, the review may offer guidance on selecting or designing health coaching models that are not only effective but also feasible and acceptable across diverse care settings.

Findings from this systematic review will be disseminated through publication in a peer-reviewed journal and presentation at relevant academic and clinical conferences. Results will also be shared with stakeholders involved in diabetes care and digital health implementation to support evidence-informed decision-making regarding the development and scaling of health coaching interventions for T2D.

### Conclusions

To conclude, based on current literature, our understanding is that no prior systematic reviews have fully examined the feasibility and active components of existing health coaching interventions exclusively for T2D self-management. Thus, this review will synthesize evidence on the effectiveness of health coaching interventions for T2D, with HbA_1c_ as the primary outcome. By examining intervention components and delivery modalities, it will inform the design of effective and sustainable health coaching interventions.

## Supplementary material

10.2196/71383 Multimedia Appendix 1 OVID search strategy.

10.2196/71383 Checklist 1 PRISMA-P checklist.

10.2196/71383 Checklist 2 CASP checklist.

## Abbreviations

AI: artificial intelligence
CASP: Critical Appraisal Skills Program
FF: face-to-face
GRADE: grading of recommendations, assessment, development, and evaluations
HbA_1c_: glycated hemoglobin
MeSH: medical subject headings
PRISMA: Preferred Reporting Items for Systematic Reviews and Meta-Analyses
PRISMA-P: Preferred Reporting Items for Systematic Review and Meta-Analysis Protocols
RC: remote coaching
ROBINS-I: Risk of Bias in Nonrandomized Studies of Interventions
T2D: type 2 diabetes
WHO: World Health Organization
