# Supplementary material for: Exploring Components and Feasibility of Health Coaching Interventions for Self-Management of Type 2 Diabetes: Protocol for a Systematic Review
Source: JMIR Res Protoc. 2026 Mar 31;15:e71383. doi: 10.2196/71383 (PMC13038180; doi:10.2196/71383)
Supplement: Multimedia Appendix 1 [file resprot-v15-e71383-s001.docx]

**Ovid Database search strategy for Medline**

| **Domain** | **Line** | **Terms** | **Search words** | **Results** |
| --- | --- | --- | --- | --- |
| Disease/  population | 1 | MeSH | Exp Diabetes Mellitus, Type 2/ |  |
| Intervention | 2 |  | Exp Mentoring/ |  |
| Study Design | 3 | MeSH | Exp controlled clinical trial/ or exp clinical trials as topic/ or exp randomized controlled trial |  |
|  | 4 | MeSH | Exp Interviews as topic/ or exp Qualitative research/ or exp focus groups |  |
|  | 5 |  | Line 3 OR 4 |  |
|  | 6 |  | Combine All MeSH results |  |
| Disease/  Population | 7 | Keywords | Diabet* mellitus type 2 OR T2D OR non-insulin dependant diabet* OR adult onset diabet* |  |
| Intervention | 8 | Keywords | Health coach* OR health coach* intervention OR motivational interviewing OR behaviour change intervention OR patient coach OR lifestyle coach |  |
| Study Design | 9 | Keywords | Randomi?ed controlled trial OR RCT OR controlled trial OR clinical trial |  |
|  | 10 | Keywords | Qualitative research OR qualitative interview* OR focus group OR thematic analysis |  |
|  | 11 |  | Line 9 OR 10 |  |
|  | 12 |  | Combine All Keyword results |  |
|  | 13 |  | Mesh OR keyword results |  |
